# Management of Hyperglycemia and Diabetes in Orthopedic Surgery

**DOI:** 10.1007/s11892-017-0839-6

**Published:** 2017-03-06

**Authors:** Funke Akiboye, Gerry Rayman

**Affiliations:** 1 The Diabetes and Endocrine Center, The Diabetes Foot Clinic and Diabetes Research Unit, Ipswich Hospital and University of Birmingham, Suffolk, IP4 5PD UK; 2 Ipswich Hospital and University of Birmingham, Birmingham, B15 2TT UK

**Keywords:** Diabetes mellitus, Hyperglycemia, Peri-operative glucose, Orthopedic surgery, Arthroplasty

## Abstract

An increasing number of orthopedic operations are being carried out in an older population in whom the prevalence of diabetes is dramatically increasing. People having surgery with diabetes and hyperglycemia are at increased risk of post-operative complications. The peri-operative risks have been well demonstrated for cardiac surgery and, more recently, for orthopedic surgery. This paper considers the issues surrounding orthopaedic surgery in patients with diabetes and the significance and management of hyperglycemia in the peri-operative period.

## Introduction

The global prevalence and incidence of diabetes is steadily rising in all populations and at the current rate the International Diabetes Foundation (IDF) estimates a prevalence of 9.9% worldwide by 2030. With population growth, this represents a 50.7% increase in people affected over a 19-year period [1]. A person with diabetes is more likely to require surgery than a person without and this is particularly notable for orthopedic surgery, which has seen an overall increase in procedures in this population as a whole [2].

Observational studies show that in the surgical patient, diabetes is associated with a higher rate of peri-operative complications such as need for transfusion, pneumonia, delayed discharge, surgical site infections, and in-hospital mortality [3]. These poorer outcomes are in part due to higher rates of co-morbid conditions such as ischemic heart disease, renal impairment, and hypertension in patients with diabetes [4]. Dysglycemia, which encompasses hyperglycemia, hypoglycemia, stress-induced hyperglycemia, and excessive glucose variability, is increasingly observed and associated with poorer post-operative outcomes even in those without a prior diagnosis of diabetes. In fact, several studies have shown post-operative complications occur more frequently in people with stress-induced hyperglycemia with no prior diagnosis of diabetes than in those with diabetes [5–7].

## Effect of Surgery on Glucose Levels

Surgery and anesthesia elicit a stress response that produces marked neurophysiological changes with release of adrenaline, noradrenaline, cortisol, glucagon, and growth hormone. This increase in counter-regulatory hormones and cytokines raises glucose levels and increases insulin resistance. In susceptible patients, this may result in significant hyperglycemia [8]. Additionally, elements of the surgical process such as disturbed eating patterns due to fasting or post-operative nausea, omission of insulin or hypoglycemic medication, and complications such as wound infection can all contribute to dysregulation of glucose homeostasis in the peri-operative period. The resulting raised, low, or erratic glucose levels have all been associated with poor outcomes.

## Effect of Glycemia on Outcomes for Orthopedic Surgery

Hyperglycemia impairs leucocyte function causing immunocompromise with consequences for superficial and deep tissue infection as well as overall mortality [9].

The detrimental effects of suboptimal glycemic control on surgical outcomes and post-operative complications have been demonstrated across surgical specialities. Within orthopedics, the subspecialties of spinal surgery, arthroplasty, and trauma surgery have been most extensively studied.

### Surgical Site Infection

Surgical site infection is the most common hospital acquired infection and is associated with worse functional outcomes and increased amputation rates in patients undergoing orthopedic surgery [10, 11]. Rates of surgical site infection are significantly increased in patients with diabetes, most notably in those with suboptimal glycemic control. Preoperative HbA1c levels above 7.0% have been found to be associated with higher rates of surgical site infection for thoracic and lumbar spinal instrumentation surgery (35.3% compared with 0.0%) [12]. The incidence of surgical site infections has also been linked to peri-operative hyperglycemia in people without a prior diagnosis of diabetes. In this group, stress-induced hyperglycemia >200 mg/dL (11.1 mmol/l) is an independent risk factor for surgical site infection at 30 days (OR 3.2, 95% CI: 1.3–7.8) even after adjustment for open fractures [13]. Following trauma, a 7-fold increase in peri-operative infections has been seen with peri-operative hyperglycemia >220 mg/dL (12.2 mmol/l, *P* = 0.0056) [6].

### Peri-prosthetic Infection

Although uncommon, occurring in around 1% of arthroplasties, peri-prosthetic infection is one of the most devastating orthopedic complications for patients and surgeons and may require further surgery to treat effectively [14]. No significant association has been demonstrated between peri-operative HbA1c and peri-prosthetic infection at the hip or knee; however, both pre- and post-operative hyperglycemia have been associated with this serious complication [15•, 16]. Morning post-operative hyperglycemia >140 mg/dL(7.8 mmol/L) is associated with a 3-fold increased risk of peri-prosthetic infection (27/285 compared with 20/582) [17]. In a series of 1565 primary knee arthroplasties, Jämson and colleagues showed a 4-fold increase in people with a peri-operative glucose >6.9 mmol/L compared with those with a normal reading of <6.1 mmol/L [18].

### Other Complications, Length of Stay, and Morbidity

It is apparent that glycemia correlates with multiple poor post-operative outcomes in orthopedic surgery, including mortality. Raised HbA1c is associated with increased length of hospital stay and is a predictor of risk for pulmonary embolism following orthopedic surgery [19]. Pre-operative HbA1c over 6.5% has been shown to be a significant risk factor for surgical outcome with a poor post-operative recovery rate following cervical laminoplasty (OR 2.6, *P* = 0.02) [20]. Undergoing primary joint arthroplasty with an HbA1c above 7.0% carries an increased mortality (HR 1.3, *P* = 0.01) [15•].

### Glycemic Management and Surgical Outcomes

Despite the above associations, the key question is whether treating hyperglycemia reduces complications and improves these outcomes. There are no randomised studies in orthopedic surgery; however, one observational study by Agos et al. demonstrated that implementation of an evidence-based standard to control hyperglycemia reduced the rate of surgical site infection in people undergoing hip and knee replacement surgery [21••].

Treating hyperglycemia in the peri-operative period has been shown to reduce complications in other surgical disciplines. Trussel and colleagues demonstrated reductions in surgical site infection with tight peri-operative glycemic control in people having coronary artery bypass grafting (CABG) surgery [22]. Similarly, Furnary and colleagues showed reductions in mortality and deep infection rates by treating people with diabetes undergoing cardiac surgery; the lowest rates seen in people targeted under 150mg/dL (8.3 mmol/L) [23]. In general surgery, Umpierrez showed that lower rates of hyperglycemia, using a basal bolus regime, was associated with reduced incidence of wound infection, pneumonia, bacteremia, and respiratory and acute renal failure [24]. These limited data have provided the basis for the American Association of Clinical Endocrinologists (AACE) and American Diabetes Association (ADA) guidelines, the Joint British Diabetes Society (JBDS) guidelines, and others all advocating treatment of hyperglycemia for hospitalized patients peri-operatively [25–27]. The target glucose ranges for these guidelines are summarized in Table 1.

**Table 1:** Peri-operative glucose targets in national guidelines

Group	Target (noncritically ill patients)	Publication year
Joint British Diabetes Societies For NHS Diabetes (26)	6–10 mmol/L (108–180 mg/dL) target 4–12 mmol/L (72–216 mg/dL) acceptable	2012
AACE/ADA/Endocrine society (25)	<140 mg/dL (7.8 mmol/L) and a random BG of less than 180 mg/dL (10.0 mmol/L) Consider lower targets in those with previously tight control	2009
Canadian Diabetes Association (27)	Fasting 5.0-8.0 mmol/L (90–144 mg/dL) Random <10 mmol/L (if safely achievable)	2013
The Association of Anaesthetists of Great Britain and Ireland (56)	6–10 mmol/L (108–180 mg/dL) target (intra-operatively) 6–12 mmol/L (108–216 mg/dL) acceptable	2015

## The Effect of Diabetes and Its Complications on Peri-operative Risk in Orthopedics

Diabetes irrespective of glycemia is associated with greater surgical risk because of the higher incidence of co-morbid conditions, including obesity, sleep apnea, hypertension, in addition to the micro- and macrovascular complications that are associated with the condition. In people with diabetes, surgery at the hip, knee, ankle, and elbow is associated with higher rates of post-operative infection, need for transfusion, pneumonia, urinary tract infection, length of stay, non-routine discharge, and in-hospital mortality [28, 29].

The condition of diabetes, in a recent meta-analysis, has shown an increased surgical site infection rate from pooled US data with OR 1.26 (95% CI 1.01–1.66) for arthroplasty and 1.6 (CI 1.10–2.32) for spinal surgery after accounting for impact of hyperglycemia and adjusting for BMI [30]. Interestingly, diabetes treated with insulin has been linked to increased 30-day readmission rates for people undergoing arthroplasty [31]. This may relate to disease chronicity, which has been associated with poor outcomes in cervical laminoplasty for people with 10 or more years duration of diabetes [20].

Neuropathy and vascular insufficiency, which may complicate diabetes, presents challenges for lower limb and particularly foot and ankle surgery, for which a modified approach is taken. Subsequent hospital-acquired foot ulceration in high risk feet can take several months to heal and superadded infection may complicate these ulcers, increasing the risk of peri-prosthetic infection and amputation [32].

### Diabetic Cardiovascular Disease and Surgery

A 2- to 3-fold increased prevalence of cardiovascular disease in people with diabetes makes surgery a higher-risk undertaking for this group [33, 34]. Hyperglycemia causes an osmotic diuresis, and the resulting hypovolemia and electrolyte disturbance may be further exacerbated by nausea and vomiting caused by anesthetic agents. The result may be tachycardia, hypokalemia, and hypomagnesemia with the resulting arrhythmias contributing to the increased peri-operative cardiovascular mortality in these patients [35].

As cardiac ischemia may be ‘silent’ in people with diabetes, a baseline ECG should be carried out in all people with diabetes prior to surgery. The threshold for cardiology referral and further investigations should be determined locally but based on the relevant national guidelines.

The following sections deal with peri-operative clinical assessment of elective surgery from pre-hospitalization through to discharge including pre-operative assessment, hospital admission, theater and recovery, and the post-operative period. The considerations of pumps, steroid-induced hyperglycemia, and emergency surgery are also discussed.

## Prehospitalization Assessment

### Surgical Outpatients

It is clear from the previous discussion that the orthopedic surgeon’s decision to proceed to surgery should include some assessment of the surgical risk to the patient with diabetes taking into account their diabetic complications and other comorbidities. High risk patients should be highlighted prompting early review, usually in a pre-assessment clinic to assess and optimize medication, glycemia, and blood pressure control prior to surgery. Specialist referrals for detailed assessment and optimization of diabetes or its cardiac or renal complications can be initiated at this stage.

One of the aims throughout the peri-operative process is to reduce the glycemic variability that may result from meal disruption and surgery itself.

Glycemic variability can be described as the degree to which glucose values fluctuate between peaks and troughs for an individual. There is no universally agreed ideal method to calculate it at present. It has emerged as a contributing factor for macro- and microvascular complications in the outpatient setting in people with both type 1 and type 2 diabetes. However, its impact in hospitalized patients has yet to be fully established. There are studies highlighting its importance in the critical care and peri-operative setting. A retrospective study by Egi et al. looked at 7049 critically ill patients with glucose measurements taken at least 4-hourly. Glucose variability was an independent risk factor in predicting mortality in ICU and hospitalized patients with an OR of 1.28 per mmol/LSD for variability and 1.21 per mmol/L for glucose level, hinting that glycemic variability is at least as important as hyperglycemia this patient group [36]. Although there are no prospective randomized trials demonstrating the impact of glycemic variability on orthopedic complications or that reducing variability improves outcomes, it is sensible to aim to limit glycemic variability until evidence to the contrary emerges.

In order to minimize glycemic variability, it is advised that patients with diabetes are prioritized on morning or afternoon lists, thereby limiting the period of fasting and disruption to normal meal times. JBDS guidelines suggest 95% of patients with diabetes should be on the first third of elective lists with avoidance of elective evening surgery altogether [26]. The regimen used to manage inpatient hyperglycemia also impacts on glucose variability, which is discussed later in this article.

**Fig. 1:**
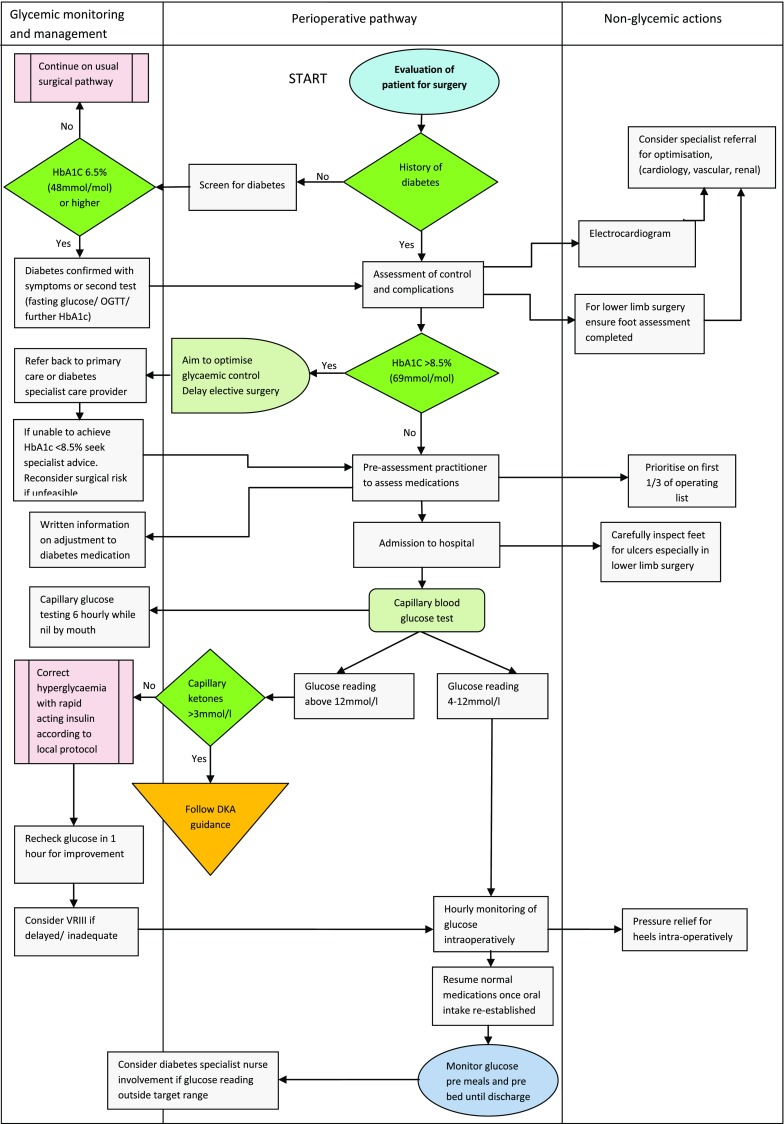
Orthopedic peri-operative pathway decision tool

Accessible electronic systems can facilitate priorities in care pathways by highlighting those people with diabetes. A flag initiated from first contact and visible throughout the peri-operative journey can be used to prompt actions such as priority list position. The glycemic and non-glycemic considerations throughout the peri-operative period are highlighted in Fig. 1.

## Preoperative Assessment

### Screening for Diabetes

Since a suspected 1% of the UK population and up to 3% of people in the US are thought to have undiagnosed diabetes, it is increasingly common for diabetes to be detected on pre-operative bloods on the day of surgery. However, screening for diabetes in the pre-assessment period is not routine, but should be considered. One prospective study of people undergoing elective noncardiac surgery found hyperglycemia in over 25% (118/493) of those without a prior diagnosis of diabetes on the morning of surgery [37].

Failure to identify and manage diabetes and hyperglycemia pre-operatively has been shown to increase the risk of complications with higher requirement for resuscitation, re-intubation, longer post-operative ventilation, and increased mortality [7, 38]. Indeed, studies to date suggest that the risk in those not known to have diabetes and those with stress or pre-operative glycemia is several fold greater than those with diabetes.

Guidelines at present do not recommend diabetes screening of patients being assessed for all types of elective surgery. The National Institute of Clinical Excellence (NICE) suggests testing for diabetes in people admitted to hospital at risk of the disease; however, this is generally limited to emergency admissions [39]. The ADA recommends screening for people over the age of 45 years and earlier in those with BMI > 25 kg/m^2^ with age adjustment for higher risk ethnic groups [40]. However, for elective orthopedic surgery, one meta-analysis suggests that it should be considered to minimize complications in the peri-operative period [41••]. HbA1c is increasingly being measured for this purpose with a value of 48 mmol/L(6.5%) or more diagnosing the condition in a symptomatic patient [42]. As the prevalence of diabetes rises, there is a potentially huge economic burden, which may result from adopting screening for diabetes in surgical pathways. If screening were adopted for all surgical pathways, this expense would need to be justified with randomized controlled trials. Although the amalgamated research in all non-cardiac surgery may be insufficient to recommend screening in national guidelines, it should be adopted in orthopedic surgery and vascular surgery based on current evidence [41••].

### Glycemic Assessment of the Diabetic Patient

Measurement of HbA1c highlights those with poorly controlled diabetes allowing optimization of glycemic control prior to surgery. It may be necessary to delay elective surgery to facilitate this.

There is no evidence-based guideline published that precludes surgery above a particular value for HbA1c; however, most guidelines advise below 8–9% (68–75 mmol/mol) for elective orthopedic surgery as a safe target [26]. In view of the complications correlated with raised HbA1c in orthopedics, some institutions may opt for lower pre-operative values than guidelines recommend. However, data suggests a significant proportion of patients scheduled for arthroplasty will take over 6 months to attain the guideline target HbA1c and for others it may not be feasible [20, 43].

### Complications and Comorbidities

The complications of diabetes should be actively assessed and optimized in view of the associated peri-operative risk they contribute. For patients undergoing foot and ankle surgery, it is essential to undertake a preoperative neurovascular assessment of the feet in addition. Neuropathy is associated with increased surgical site infection rates even in those without diabetes. High risk feet can be identified without any equipment using a quick and simple bedside tool, the ‘Ipswich touch test’, prompting pressure relief as soon as convalescent [44]. Pre-operative revascularization may be necessary in some people with significant peripheral vascular disease.

The pre-operative workup for people with diabetes undergoing surgery is covered in detail in both the Joint British Diabetes Societies and ADA guidelines and the additional management considerations relating to diabetes should be incorporated into local orthopedic pathways and proformas to prevent omission of these elements during the peri-operative period. Clear written instructions on the alterations to medications according to local guidelines should be given to patients at pre-assessment.

A self-reporting checklist for patients with diabetes to complete can alert staff to potential problems for further attention.

## Hospital Admission

### Glycemic Control and Monitoring

The aims of peri-operative glycemic control are avoiding hypoglycemia, marked hyperglycemia, electrolyte disturbance, and the diabetic emergencies of hyperosmolar hyperglycemic state (HHS) and diabetic ketoacidosis (DKA). The target range that is most effective in achieving these aims remains a matter of debate.

The NICE-SUGAR trial and a series of studies challenged the dogma for tight glycemic control outside of the critical care or cardiology setting with increased mortality attributed to higher rates of hypoglycemia [45]. The emphasis is increasingly on adapting the glycemic target to the individual patient and their circumstances. Published guidelines focus on safe rather than tight control with JBDS promoting glucose targets between 6 and 10 with 4–12 mmol/L as an acceptable range. A selection of differing peri-operative targets by country are displayed in Table 1.

Close monitoring in the peri-operative period is required to detect and manage glucose excursions and is advised before meals or 4–6 hourly in patients who are not eating. Blood tests on admission should include a laboratory glucose or capillary blood glucose with point of care testing. There should be clear local policies to optimize glycemic control in the hospital throughout the peri-operative period with methods governed by the individual patient circumstances, the policy and resources of the institution, and clinician’s judgement.

### Fasting and Enhanced Recovery

Prolonged fasting results in increased insulin resistance in both those with and without diabetes. Higher insulin resistance is associated with poor wound healing, higher complication rates, and increased length of hospital stay. The degree of insulin resistance is greater in larger or more complex operations and those with greater blood loss and through metabolic pathways, it contributes to hyperglycemia [46]. A meta-analysis has shown insulin resistance may be attenuated by half with administration of oral or IV glucose, associated with reductions in length of stay [47]. Pre-operative oral carbohydrate treatment has been widely adopted as part of an enhanced recovery program; a multimodal approach to peri-operative care, which includes early mobilization, minimization of the fasting period, and optimizing pain relief. Enhanced recovery programmes have been shown to reduce length of stay and post-operative complications, most notably in colorectal surgery [48].

Although enhanced recovery programmes show improved post-operative outcomes, there is limited evidence demonstrating the benefits of pre-operative carbohydrate loading specifically within these programmes. The impact of carbohydrate loading in improving a range of post-operative outcomes has been examined in a Cochrane review of 27 randomized controlled trials, including 4 orthopedic studies. The authors of this review concluded that preoperative carbohydrate loading offers small reductions in length of stay; however, complications and well-being are not significantly impacted [49].

There is a paucity of data on the potential impact of pre-operative carbohydrate loading on those with diabetes. A small case controlled study by Gustaffson et al. found no delay in gastric emptying following a carbohydrate drink for subjects with type 2 diabetes compared with healthy volunteers. In this study, peak glucose concentrations occurred later and were higher (13.4 ± 0.5 compared with 7.6 ± 0.5 mmol/l; *P* ≤ 0.01) [50].

With the aforementioned concerns about increased complication rates related to peri-operative hyperglycemia, the administration of carbohydrate drinks in people with diabetes requires further study before it is implemented for this group, particularly in orthopedic surgery. The exclusion of people with insulin treated diabetes from these programmes [51] may limit further detailed study of this group.

### Immobility and Pressure Ulcers

Pressure ulcers are a problem in surgery, causing pain, reduced quality of life, and prolonged hospital admission; diabetes can increase the risk of pressure ulcers 3-fold. Pressure ulcers following hip surgery can have devastating consequences with associated complication rates ranging from 16 to 46% and an increased mortality rate of 27% [52].

Prevention is the most effective way to approach this problem with simple inspection found to be more effective than currently advocated scoring systems. As the majority of these ulcers develop in-hospital, often on the day of surgery, it is vital that preventative measures commence on admission [53].

The period of immobility should be minimized and feet should be carefully inspected preoperatively for ulceration, which may act as a port for infection that people with diabetes may not self-report due to neuropathy. Multidisciplinary team involvement is crucial in those who develop ulceration as resultant hyperglycemia in those with infected ulcers can create a vicious cycle by delaying wound healing.

## Theater and Recovery

### Glucose Targets and Glucose Management

There are limited data examining the effect of intra-operative glycemic control on post-operative outcomes with available studies carried out in cardiac surgery. Doenst et al. showed an intra-operative peak glucose reading >20 mmol/L(360 mg/dL) to be an independent risk factor for poor post-operative outcome and mortality in people with diabetes (OR, 1.20; CI, 1.08–1.32) and without diabetes (OR, 1.12; CI, 1.06–1.19; per mmol/L increase in glucose) [54]. Ouattara et al. found poor intra-operative glycemic control, defined as 4 or more consecutive blood glucose values over 11.1 mmol/L(200 mg/dL), to confer a higher risk of cardiac and noncardiac post-operative complications [55]. The Association of Anesthetists of Great Britain and Ireland (AAGBI) advise an intra-operative glucose target of 6–10 mmol/L with a peak up to 12 mmol/L for less well controlled patients. Ketone testing is advised with intra-operative glucose readings over 12 mmol/L[56].

Glucose testing remains central to maintaining glycemic control and should be carried out prior to induction of anesthesia, then hourly for operations of over 2 hours. For prolonged fasting with more than 1 missed meal or operations over 3 hours, an insulin infusion may be required. In these instances, a glucose, insulin, potassium (GIK) regime may be used, where the 3 elements are combined in a single bag for infusion. However, many institutions favor the variable rate intravenous insulin infusion (VRIII), where insulin and glucose with or without potassium are delivered as separate infusions with titration of the insulin every 1–2 hours. This requires close monitoring and regular adjustment as potentially dangerous glucose excursions can occur when the infusions are mismatched. In a small study looking at patients undergoing cardiothoracic surgery, the VRIII was found to offer more stable glycemic control than the GIK regime [57]; however, there is little evidence in support of one method over the other, and local policy should be followed. VRIII should commence at least 2 hours prior to surgery to allow time for abnormal glucose readings to stabilize.

For people on a long-acting basal insulin, local policies usually advise continuation of the basal insulin with the variable rate insulin infusion. This facilitates transition back to the usual regime when oral intake is reinstated. If the basal insulin is discontinued preoperatively, it must be given 30 to 60 minutes prior to any intravenous insulin being taken down because of the short half-life of intravenous insulin in order to reduce the risk of diabetic ketoacidosis.

### Pumps

For people on continuous subcutaneous insulin infusion (insulin pumps) undergoing day case surgery, there is evidence that this may be safely continued intra-operatively for up to 3 hours whilst maintaining safe glycemic control post-operatively with the support of the specialist endocrine team [58]. Alternatively, subcutaneous basal insulin may be used at a dose equivalent to the background insulin dose administered over 24 hours on the pump.

There are a few considerations when planning to continue insulin pumps intra-operatively. It is generally recommended that pumps be removed for X-ray, CT, or MRI scanning; however, covering the pump with a lead shield may be sufficient for X-rays. The need for imaging intra-operatively is often pre-empted and subcutaneous insulin may be a good alternative to pump use in these circumstances. Additionally, electro-cautery and the presence of flammable anesthetic mixtures with oxygen or nitrous oxide in the operating room may affect the function of the pump, so the manufacturer’s guidance should be reviewed and followed [59].

### Foot Protection

Pressure damage leading to subsequent ulceration may begin within a few hours of sustained pressure; therefore repositioning has been advised at least every 2 hours intra-operatively where possible. An array of pressure relieving devices are available, from foam mattresses to inflatable heel supports, which can reduce the development of pressure ulcers by up to 70%. However, there is no single, recommended device [60] and most orthopedic units employ gel pads or inflatable air-boots. Limb surgery is discounted since the operated limb is mobile intra-operatively.

### Antibiotics

The role for peri-operative antibiotic prophylaxis in joint arthroplasty is well documented [61, 62]. People who are morbidly obese have differences in antibiotic pharmacodynamics and pharmacokinetics, making their efficacy less predictable in these people. Obesity is associated with increased rates of surgical site infection and studies by Dowsey and Choong have shown it to be an independent risk factor for peri-prosthetic infection at both hip and knee [63, 64]. As a significant proportion of people with type 2 diabetes will be obese, weight-adjusted dosing should be considered.

A few antibiotics have been studied in this population, including aminoglycosides, vancomycin, daptomycin, and linezolid. Weight-adjusted antibiotic dosing is not widely practiced in the UK or recommended in the guidance; however, the additional risk of obesity is considered in the dosing advice in Australian and US guidance. The American Association of Surgeons (AAOS) recommend weight-adjusted dosing for a range of antibiotics with a double dose of 2g cephalexin for patients over 80kg [65].

## Post-operative Period

### Managing Medication

Post-operatively, diet and usual diabetes medications should be restarted as soon as possible; however, there are a few oral agents for which clinical circumstances should be considered before reinstating. Metformin carries a risk of lactic acidosis, particularly in those with renal insufficiency. It should be omitted in patients who develop acute kidney injury until renal function returns to near baseline or eGRF is above 30 mL/min/1.73m^2^. It should also be withheld in sepsis, congestive cardiac failure, and significant hepatic impairment, all of which may be associated with hypoxia increasing the risk of lactic acidosis. Thiazolidinediones such as pioglitazone should not be restarted if significant fluid retention or congestive cardiac failure have developed or when there are liver function abnormalities. In patients who have not resumed a normal diet, sulphonylureas such as gliclazide may be withheld because of their insulin secretory effects with potential for causing hypoglycemia. A smaller dose may be initiated and titrated up as oral intake returns to normal. A cautious re-introduction should also be considered where there has been a kidney injury, as reduced renal excretion can further augment and prolong hypoglycemia. Recent studies have demonstrated the safety and efficacy of DPP4 inhibitors, the gliptins, in people with mild to moderate hyperglycemia (200 mg/dl) [66]. In view of the potential problems with sulphonylureas, it is likely that the use of these agents will become more widespread.

Insulin infusions should continue until eating has resumed. On recommencing usual insulin treatment, the subcutaneous insulin dose should be administered 30 minutes prior to discontinuing the intravenous infusion, which has a 5–10 minute half-life to minimize the risk of DKA and glucose excursions.

### Treating Hyperglycemia

Following surgery, management of hyperglycemia remains important both for people with diabetes and those with stress-induced hyperglycemia. Due to its breadth of usability, insulin remains the main agent for controlling hyperglycemia for in-hospital patients, and can be used regardless of comorbidities or altered clinical states such as impaired renal function and decompensated cardiac failure. Correction doses of insulin may be used in insulin-naïve people, but titrating doses of subcutaneous insulin referred to in the US as sliding scale are not advised. A recent meta-analysis demonstrated these corrective subcutaneous doses do not offer tighter glycemic control for hospitalized patients and are associated with higher rates of hyperglycemia than a range of other regimes without any significant reduction in length of hospital stay [67]. Due to these observed increases in glucose variability, the subcutaneous ‘sliding scale’ should not be used as the sole method of glycemic control, but may still be used to supplement other regimes.

### Hypoglycemia

Hypoglycemia is unpleasant for patients and associated with higher mortality rates and longer hospital length of stay. It is important to remain vigilant to this risk and check capillary glucose in patients in whom an altered mental state may otherwise be attributed to delirium or drowsiness secondary to concomitant analgesia following surgery. With its associated risks, hypoglycemia can be a barrier to intensifying glucose treatment.

In the case of missed meals or interrupted nutrition, nurse driven protocols and automated management decision tools for the omission of sulphonylureas and prandial insulin should be implemented locally to reduce the risk of iatrogenic hypoglycemia and standardize care.

### Specialist Teams

It is increasingly common for elderly orthopedic patients to be managed by an orthogeriatrician to focus on the medical aspects of care across the peri-operative period. While this has improved the overall medical treatment, the specific impact for quality of care for people with diabetes has not been studied. Specialist diabetes nursing teams often have a lower threshold for discharge and can considerably reduce length of stay for elective procedures with significant cost-saving implications [68]. Their input at key points in the peri-operative pathway has been shown to be beneficial, and clear pathways for pre-operative referral and post-operative involvement should be developed locally.

## Special Circumstances

### Steroids

Steroid injections are an important adjunct in managing musculoskeletal diseases. Intra-articular injections are most frequently carried out for osteo-arthritis [69], with the knee joint the most common site of injection, followed by shoulder, wrist, ankle, and elbow joints [70]. This route allows symptomatic relief whilst minimizing systemic corticosteroid effects. However, due to concerns over peri-prosthetic infection, their use is often avoided within 3 months of elective surgery [71].

Synthetic steroids mimic endogenous glucocorticoid, binding to the nuclear glucocorticoid receptor, affecting transcription of anti-inflammatory mediators. These effects may last for months. The effects of intra-articular steroid injections on the hypothalamic-pituitary-adrenal axis have been most widely studied [72], but they may also affect glucose metabolism in a similar manner to oral steroids. This effect is exerted in a number of ways from increased insulin resistance to direct effects on pancreatic beta cell function. The resulting hyperglycemia may be asymptomatic or result in osmotic symptoms and fatigue. It often resolves spontaneously, but if persistent after steroid withdrawal, it is termed steroid induced diabetes.

The few studies examining the effect of intra-articular steroid injection on glycemic control suggest that there are only short-term effects, which vary depending on the site and type of injection [73, 74]. While the hyperglycemic effects are generally not sustained long enough to affect injection site elective orthopedic surgery due restrictions imposed by infection risk, it is worth noting that there may be an impact on glucose for up to 5 days following injection [73]. Therefore, people with previously controlled type 2 diabetes should have home glucose monitoring if elective surgery of another joint is planned with 2 weeks of steroid injection or closely monitored if emergency surgery is required.

The JBDS advise measuring glucose at least once daily, preferably prior to the evening meal, in those given steroids not known to have diabetes and if above 12 mmol/L(216 mg/dL), treating to maintain glucose levels between 6 and 10, accepting a pragmatic range of 4–12 mmol/l. They suggest HbA1C be measured in patients at high risk of steroid-induced diabetes, and a baseline level should be taken in those with pre-existing diabetes prior to commencement of steroids [75].

It is recommended to increase the frequency of glucose monitoring to 4 times a day and to commence treatment for 2 consecutive glucose readings above 12 mmol/L. For hyperglycemia resulting from intra-articular steroid injection, as with multiple daily steroid doses, oral agents are unlikely to control the hyperglycemia, and a subcutaneous insulin regime is preferred. A pragmatic approach would be to commence basal insulin and titrate this according to glucose values. In this case, a morning NPH insulin such as humulin I or insulatard is suggested, starting at 10 units daily titrating up by 10–20% every 24 hours until glucose readings are on target [75]. The local community diabetes team may be contacted for further advice and support.

## Emergency Surgery

People with diabetes are more likely to require emergency orthopedic procedures for a number of reasons. Hypoglycemia and peripheral neuropathy increase the likelihood of falls, and bone mineral density is affected, particularly in those with type 1 diabetes, increasing the fracture risk further. The orthopedic surgeon may also be involved in the management of diabetic foot emergencies depending on the local service setup.

In the emergency setting, glycemic control remains crucial to reducing post-operative complications; however, the clinical situation restricts the time to fully optimize patients. In these settings, intravenous insulin given as a variable rate infusion remains the most effective way to achieve normoglycemia in the peri-operative period. The local protocol often advises continuation of the usual background insulin for those on basal insulin to facilitate prompt return to a normal regime postoperatively.

Where possible, DKA should be treated as a priority and the fluid and electrolyte disturbances of HHS should be reduced or corrected prior to surgery.

## Conclusions

People with diabetes require particular consideration in planning orthopedic surgery. It can be difficult to put guidelines into practice across the multistep peri-operative process; however, significant improvements in care, with patient benefits and cost saving implications are possible. Specialist in-patient teams who can examine local processes, along with design and implementation of clear local pathways across the surgical process are key in minimizing the harm to this vulnerable group of patients.
